# Multi-Nyström Method Based on Multiple Kernel Learning for Large Scale Imbalanced Classification

**DOI:** 10.1155/2021/9911871

**Published:** 2021-06-13

**Authors:** Ling Wang, Hongqiao Wang, Guangyuan Fu

**Affiliations:** Department of Information Engineering, Rocket Force University of Engineering, Xi'an, 710025, China

## Abstract

Extensions of kernel methods for the class imbalance problems have been extensively studied. Although they work well in coping with nonlinear problems, the high computation and memory costs severely limit their application to real-world imbalanced tasks. The Nyström method is an effective technique to scale kernel methods. However, the standard Nyström method needs to sample a sufficiently large number of landmark points to ensure an accurate approximation, which seriously affects its efficiency. In this study, we propose a multi-Nyström method based on mixtures of Nyström approximations to avoid the explosion of subkernel matrix, whereas the optimization to mixture weights is embedded into the model training process by multiple kernel learning (MKL) algorithms to yield more accurate low-rank approximation. Moreover, we select subsets of landmark points according to the imbalance distribution to reduce the model's sensitivity to skewness. We also provide a kernel stability analysis of our method and show that the model solution error is bounded by weighted approximate errors, which can help us improve the learning process. Extensive experiments on several large scale datasets show that our method can achieve a higher classification accuracy and a dramatical speedup of MKL algorithms.

## 1. Introduction

Real-world problems in computer vision [1], natural language processing [2, 3], and data mining [4, 5] present imbalanced traits in their data, which may be developed by the inherent properties of the data or some external factors such as sampling bias or measurement error. Unfortunately, most traditional learning algorithms are designed based on balanced data and target the overall classification accuracy, leading the minority class to be overwhelmed by the majority class. However, the minority class in these real-world problems is usually more important and expensive than the majority class.

In the past few decades, many algorithms have been proposed to solve the class imbalance problems [6–8]. The data-level methods artificially balance the skewed class distributions by data sampling [9, 10]. The algorithm-level methods lift the importance of minority instances via the modification of existing learners [11, 12]. However, there usually exist complex nonlinear structures in these real-world imbalanced data. In this case, the extensions of kernel methods for the class imbalance problems have been proven very effective [13–15]. In [16], Mathew et al. overcome the limitations of the synthetic minority oversampling technique (SMOTE) for nonlinear problems by oversampling in the feature space of the support vector machine. In [17], a kernel boundary alignment algorithm is proposed to adjust the class boundary by modifying the kernel matrix according to the imbalanced data distribution. The kernel-based adaptive synthetic data generation (KernelADASYN) for imbalanced learning is proposed in [18], which uses kernel density estimation (KDE) to estimate the adaptive oversampling density. However, with the development of data storage and data acquisition equipment, the scale of data continues to grow. The existing kernel-based class imbalanced learning (kernel CIL) methods suffer from serious challenges that the cost of calculating and storing a vast kernel matrix is very expensive.

A general technique for making kernel methods scalable is kernel approximation, of which the Nyström method is the most popular one [19]. The Nyström method constructs a low-rank approximation of the original kernel matrix from a subset of *l* ≪ *n* landmark points, where *n* is the data size. Computationally, it only needs to decompose a smaller matrix (denoted as *W* ∈ *ℝ*^*l*×*l*^). However, according to the approximation error bound $O\left(n/\sqrt{l}\right)$ for the Nyström method in [20], there is a trade-off between accuracy and efficiency. The more landmark points sampled provide improved approximation accuracy but require more computing resources, which results in the rapid expansion of the subkernel matrix *W* as the data size increases and seriously affects the efficiency of the Nyström method.

Some works study the efficacy of a variety of fixed and adaptive sampling schemes for the Nyström method. For example, Musco et al. presented a new Nyström algorithm based on recursive leverage score sampling, which runs in linear time in the number of training points [21]. An ensemble Nyström method has been proposed to yield more accurate low-rank approximations by running mixtures of the Nyström method based on several subsets of landmark points randomly sampled [22]. However, the mixture weights of the ensemble Nyström method are defined according to the approximation error of each Nyström approximation, which may lead to the performance not as expected when applied to practical classification or regression applications. Recently, there emerges a fast and accurate refined Nyström-based kernel classifier to improve the performance of the Nyström-based kernel classifier [23]. Although the Nyström method has been studied extensively, there still exists a potentially large gap between the performance of learner learned with the Nyström approximation and that learned with the original kernel.

In this study, we propose a novel method, multi-Nyström, for large scale imbalanced classification. We incorporate the multi-Nyström method and multiple kernel learning to learn an improved low-rank approximation kernel superior to any one of each multi-Nyström approximation, where each approximation is defined by different kernel functions and subsets of landmark points. Moreover, unlike existing sampling schemes for the multi-Nyström method, our method selects subsets of landmark points according to the imbalance distribution to deal with the problem of skewed data. Without computing and storing the full kernel matrix, our method can scale to large scale scenarios. The main contributions of this study are summarized as follows:

1. We propose a multi-Nyström method to overcome the computational constraints of the Nyström method. Due to our method parallelized easily, it can generate more accurate approximates in large scale scenarios.
2. We optimize the mixture weights according to the data and the problem at the hand, so that the combined approximation kernel matrix can produce better performance. Moreover, the low-rank approximation can significantly speed up the existing MKL algorithms process.
3. We provide a stability analysis of our method, showing us the impact of kernel approximation error on the model solution and help determine the acceptable approximation error in the approximation of the kernel matrix.

The rest of this study is organized as follows.  Section 2 introduces some related concepts.  Section 3 then describes the proposed multi-Nyström approximation algorithm in detail. Experimental results and analysis compared with other algorithms are presented in Section 4. Finally, Section 5 summarizes the full work.

## 2. Related Work

### 2.1. Kernel Methods

Kernel methods such as support vector machines (SVMs) have become one of the most popular technologies of machine learning [24]. It can extend linear learners to nonlinear cases by introducing kernel trick. Consider a binary-class dataset *D*={(**x**_*i*_, *y*_*i*_)}_*i*=1_^*n*^, where **x**_*i*_ ∈ *𝒳*⊆*ℝ*^*s*^ denotes an s-dimensional vector and *y*_*i*_ ∈ {+1, −1} denotes its label. Define a nonlinear descriptor as

(1) $$\begin{array}{c} \Phi :X⟶ℋ\;x_{i}\mapsto \Phi \left(x_{i}\right). \end{array}$$

The input data are mapped to a high-dimensional or even infinite-dimensional feature space, and the inner product in the feature space is calculated implicitly through the kernel function defined in the input space.

(2) $$\begin{array}{c} K\left(x,x^{\prime }\right)={\langle \Phi \left(x\right),\Phi \left(x^{\prime }\right)\rangle }_{ℋ}=\Phi {\left(x\right)}^{T}\Phi \left(x^{\prime }\right), \end{array}$$

where *K* : *ℝ*^*s*^ × *ℝ*^*s*^ ↦ *ℝ* is the kernel function that satisfies Mercer's theorem [25], and ℋ is the corresponding reproducing kernel Hilbert space (RKHS). *K* can simply be a classical kernel like the radial basis function (RBF) kernel. Unfortunately, the kernel matrix *K* ∈ *ℝ*^*n*×*n*^ expands quadratically with the increase of data scale. The poor scalability limits the applicability of kernel methods in large scale scenarios.

### 2.2. Multiple Kernel Learning

Due to different kernels corresponding to different similarity concepts or using features from different views, MKL can obtain more complete representations of the input data by combining multiple kernels. In MKL, each instance (**x**_*i*_, *y*_*i*_) is mapped into different feature spaces by a series of descriptors [26]:

(3) $$\begin{array}{c} \Phi_{ℋ}\left(x_{i}\right)={\left[\sqrt{d_{1}}\Phi_{1}^{T}\left(x_{i}^{1}\right),\ldots ,\sqrt{d_{M}}\Phi_{M}^{T}\left(x_{i}^{M}\right)\right]}^{T}, \end{array}$$

where **x**_*i*_^*m*^ represents feature from the *m*^th^ view of instance **x**_*i*_, *d*_*m*_ ≥ 0, *m*=1,…, *M* is the corresponding weight, and *M* is the total number of predefined kernels. Then, substitute any dot product term with kernels:

(4) $$\begin{array}{c} K\left(x,x_{i}\right)={\langle \Phi \left(x\right),\Phi \left(x_{i}\right)\rangle }_{ℋ}\\ =\sum_{m=1}^{M}d_{m}{\langle \Phi_{m}\left(x^{m}\right),\Phi_{m}\left(x_{i}^{m}\right)\rangle }_{{ℋ}_{m}}\\ =\sum_{m=1}^{M}d_{m}K_{m}\left(x^{m},x_{i}^{m}\right), \end{array}$$

where each base kernel function *K*_*m*_(·, ·) : *ℝ*^*s*^ × *ℝ*^*s*^⟶*ℝ* is a positive definite kernel associated with an RKHS ℋ_*m*_. The purpose of MKL is to learn a resulting discriminant function of the form *f*(**x**)=∑_*m*_*f*_*m*_(**x**^*m*^)+*b* with ℋ_*m*_≔{*f*_*m*_*|f*_*m*_(**x**)=∑_*i*=1_^*∞*^*α*_*i*_*K*_*m*_(**x**^*m*^, **x**_*i*_^*m*^)}.

Based on the aforementioned definition, the seminal work in MKL proposes the following structural risk minimization framework as MKL primal problem with kernel weights on a simplex [27].

(5) $$\begin{array}{c} \underset{\left\{f_{m}\right\},b,\xi ,d}{\min}:\frac{1}{2}\sum_{m=1}^{M}\frac{1}{d_{m}}{\left\|f_{m}\right\|}_{{ℋ}_{m}}^{2}+C\sum_{i=1}^{N}\xi_{i}\\ \text{s}\mathrm{.t}\text{.}\;\begin{array}{c} y_{i}\left(\sum_{m=1}^{M}f_{m}\left(x_{i}^{m}\right)+b\right)\geq 1-\xi_{i}\\ \xi_{i}\geq 0,\;i=1,\ldots ,N\\ \sum_{m=1}^{M}d_{m}=1,\;d_{m}\geq 0,m=1,\ldots ,M, \end{array} \end{array}$$

where *C* is the regularization parameter of the error term. *ξ* is the slack variable. The L1-norm constraint on the weight vector *d* enforces the kernel combination to be sparse. We assume ‖*f*_*m*_‖_ℋ_*m*__^2^=0 whenever *d*_*m*_=0 in order to reach a finite objective. That implies if the weight of a certain kernel reaches *d*_*m*_=0, stop the optimization of *f*_*m*_ since the solution is known *f*_*m*_=0 [28].

Although MKL is an ideal candidate for combining multiview data, scalability is a key issue for MKL: (1) the computation and memory costs for maintaining several kernel matrices are heavy and (2) the computational efficiency of MKL solvers is not high.

### 2.3. Standard Nyström Method

Let *L*={**c**_1_,…, **c**_*l*_}, where **c**_*i*_ ∈ *ℝ*^*s*^ denotes a set of *l* landmark points randomly selected from *D* uniformly without replacement, *C* ∈ *ℝ*^*n*×*l*^ denotes the subkernel matrix between all instances and the landmark points, and *W* ∈ *ℝ*^*l*×*l*^ be a symmetric positive semidefinite (SPSD) subkernel matrix among the points in *L*. Then, the Nyström method uses *W* and *C* to generate a rank-*k* approximation ${\tilde{\text{K}}}_{k}$ of kernel matrix *K* for *k* ≤ *l* [20]:

(6) $$\begin{array}{c} K\approx {\tilde{K}}_{k}≔CW_{k}^{+}C^{T}, \end{array}$$

where *W*_*k*_ ∈ *ℝ*^*l*×*l*^ is the best rank-*k* approximation to *W* with respect to the Frobenius norm, that is, *W*_*k*_=argmin_rank(*V*)=*k*_‖*W* − *V*‖_*F*_, and *W*_*k*_^+^ denotes the pseudoinverse of *W*_*k*_. Given the matrix *W*_*k*_, the feature of each instance **x**_*i*_ can be evaluated as

(7) $$\begin{array}{c} \phi \left(x_{i}\right)=\sqrt{W_{k}^{+}}{\left(K\left(x_{i},c_{1}\right),\ldots ,K\left(x_{i},c_{l}\right)\right)}^{T}. \end{array}$$

Calculate the singular value decomposition (SVD) of *W* as *W*=*U*Λ*U*^*T*^, where *U* is the orthonormal and Λ=diag(*σ*_1_,…, *σ*_*m*_) is the diagonal with *σ*_1_ ≥ ⋯≥*σ*_*m*_ ≥ 0. Then, the final approximate decomposition of *K* is denoted as the following form:

(8) $$\begin{array}{c} K\approx {\tilde{U}}_{k}{\tilde{\Lambda }}_{k}{\tilde{U}}_{k}^{T},\;\text{with }{\tilde{U}}_{k}=\sqrt{\frac{l}{n}}CU_{k}\Lambda_{k}^{-1},{\tilde{\Lambda }}_{k}=\frac{n}{l}\Lambda_{k}, \end{array}$$

where Λ_*k*_ ∈ *ℝ*^*k*×*k*^ is the diagonal formed by the top *k* singular values of Λ, and *U*_*k*_ ∈ *ℝ*^*l*×*k*^ is formed by the associated singular vectors.

The total time complexity of the Nyström method is *O*(*l*^3^+*nlk*) including *O*(*l*^3^) for SVD on *W* and *O*(*nlk*) for matrix multiplication with *C* [29]. For *l* ≪ *n*, it is much lower than the *O*(*n*^3^) complexity taken by SVD on *K*.

## 3. Proposed Algorithms

### 3.1. Multi-Nyström Method

We divide the imbalance dataset *D*={(**x**_*i*_, *y*_*i*_)}_*i*=1_^*n*^ into the minority class set *D*^+^={(**x**_*i*_, +1)}_*i*=1_^*n*_+_^ and the majority class set *D*^−^={(**x**_*i*_, −1)}_*i*=1_^*n*_−_^. When there are irregularities in the imbalanced data (such as small disjuncts, overlapping, and noise [30]) and the data scale is large, applying a single kernel may make the model biased, skew, or misleading. Inspired by the MKL algorithm [31], we construct a low rank approximate multiple kernel framework as follows:

(9) $$\begin{array}{c} K\left(x,x_{i}\right)\approx \sum_{m=1}^{M}d_{m}{\tilde{K}}_{m,k}\left(x^{m},x_{i}^{m}\right),\;\text{with }d_{m}\geq 0, \end{array}$$

where ${\tilde{K}}_{m,k}$ corresponds to the rank-*k* approximation of each base kernel matrix *K*_*m*_, and *d*_*m*_ is the corresponding mixture weight. As for the Nyström method, a key aspect is the sampling scheme [32]. For reducing the sensitivity to skewness in data, we adopt the stratified undersampling of the majority class to select *M* subsets of landmark points written as *L*={*L*_*m*_}_*m*=1_^*M*^ with each *L*_*m*_={**c**_*m*,1_,…, **c**_*m*,*l*_}. The subkernel matrix between all instances and the landmark points can be expressed as

(10) $$\begin{array}{c} C=\left[C_{1},\ldots ,C_{M}\right]\in {\mathbb{R}}^{n\times Ml}, \end{array}$$

where *C*_*m*_ ∈ *ℝ*^*n*×*l*^. Then, we perform the standard Nyström method on each *C*_*m*_ independently to get a rank-*k* approximation ${\tilde{K}}_{m,k}=C_{m}W_{m,k}^{+}C_{m}^{T}$ of each base kernel matrix *K*_*m*_. Finally, by linearly combining these approximations, we can get the general form of approximation multiple kernel $\tilde{K}$:

(11) $$\begin{array}{c} \tilde{K}=\left[C_{1},\ldots ,C_{M}\right]\left[\begin{array}{ccc} d_{1}W_{1,k}^{+} & & \\ & \ddots & \\ & & d_{M}W_{M,k}^{+} \end{array}\right]\left[\begin{array}{c} C_{1}^{T}\\ \vdots \\ C_{M}^{T} \end{array}\right]. \end{array}$$

Given the mixture weight *d*_*m*_, the feature of each instance **x**_*i*_ can be evaluated as

(12) $$\begin{array}{c} \tilde{\phi }\left(x_{i}\right)=\left[\begin{array}{c} \sqrt{d_{1}W_{1,k}^{+}}{\left(K_{1}\left(x_{i},c_{1,1}\right),\ldots ,K_{1}\left(x_{i},c_{1,l}\right)\right)}^{T}\\ \vdots \\ \sqrt{d_{M}W_{M,k}^{+}}{\left(K_{M}\left(x_{i},c_{M,1}\right),\ldots ,K_{M}\left(x_{i},c_{M,l}\right)\right)}^{T} \end{array}\right]. \end{array}$$

**Figure 1:**
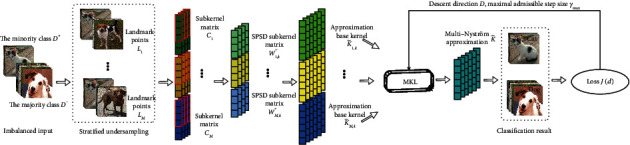
Architecture of the proposed multi-Nyström method. *M* subsets from the majority class are sampled to construct balanced landmark points and then the Nyström method is used to obtain the approximate base kernel matrices and the multiple kernel learning (MKL) algorithm is applied to optimize the mixture weights and train classifier. Finally, the trained kernel classifier based on multi-Nystrom is obtained.

Similarly, for the convenience of subsequent calculations, formula (11) can be rewritten as

(13) $$\begin{array}{c} \tilde{K}=\left[{\tilde{U}}_{1,k},\ldots ,{\tilde{U}}_{M,k}\right]\left[\begin{array}{ccc} d_{1}{\tilde{\Lambda }}_{1,k} & & \\ & \ddots & \\ & & d_{M}{\tilde{\Lambda }}_{M,k} \end{array}\right]\left[\begin{array}{c} {\tilde{U}}_{1,k}^{T}\\ \vdots \\ {\tilde{U}}_{M,k}^{T} \end{array}\right]. \end{array}$$

where ${\tilde{U}}_{m,k}\in {\mathbb{R}}^{n\times k}$, and ${\tilde{\Lambda }}_{m,k}\in {\mathbb{R}}^{k\times k}$ denotes the approximate decomposition of *K*_*m*_ obtained by (8).  Figure 1 shows the proposed multi-Nyström method and includes an optimization process of the mixture weights detailed futher in next subsection.

When the mixture weight *d*_*m*_ is fixed or known, the total time complexity of the multi-Nyström method is *O*(*Ml*^3^+*Mnlk*). Although our method requires *M* times more CPU resources than the standard Nyström method, *M* ≪ *n* is typically *O*(1) for large scale data, and our method can compute in parallel in the distributed computing environment. Moreover, the SVD on the subkernel matrix *W* is decomposed into that on *M* much smaller matrices would also accelerate the calculation process.

### 3.2. Optimization to Mixture Weights

The purpose of MKL is to learn an optimal convex combination of a series of kernels during training. Based on the aforementioned definition, we propose an approximate multiple kernel learning framework for large scale imbalanced classification by modifying the original MKL framework in [26]

(14) $$\begin{array}{c} \min J\left(d\right)\text{ such that }{\left\|d\right\|}_{1}^{2}=1,\;d_{m}\geq 0,m=1,\ldots ,M, \end{array}$$

where

(15) $$\begin{array}{c} J\left(d\right)=\left\{\begin{array}{cc} \underset{\alpha }{\min} & \frac{1}{2}\alpha^{T}Y\tilde{K}Y\alpha -e^{T}\alpha \\ s\mathrm{.t}\text{.} & \begin{array}{c} y^{T}\alpha =0\\ 0\leq \alpha \leq C, \end{array} \end{array}\right) \end{array}$$

where *α* is the Lagrange multipliers vector, and *Y*=diag(*y*_1_,…, *y*_*n*_). To avoid numerical instability caused by ill-conditioning [19], we substitute ${\tilde{K}}_{m,k}⟵{\tilde{K}}_{m,k}+\sigma I$, where *σ* is a small positive constant called jitter factor. Moreover, to calculate the inverse of the approximate matrix ${\tilde{K}}^{-1}$ and avoid storing the complete *n* × *n* matrix $\tilde{K}$, we iteratively perform the following series of operations:

(16) $$\begin{array}{c} T_{0}^{-1}=\frac{1}{\sigma }I,\\ T_{1}^{-1}={\left(T_{0}+d_{1}{\tilde{K}}_{1,k}\right)}^{-1}\\ ={\left(T_{0}+d_{1}{\tilde{U}}_{1,k}{\tilde{\Lambda }}_{1,k}{\tilde{U}}_{1,k}^{T}\right)}^{-1}\\ \cdots \\ T_{M}^{-1}={\left(T_{M-1}+d_{M}{\tilde{K}}_{M,k}\right)}^{-1}\\ ={\left(T_{M-1}+d_{M}{\tilde{U}}_{M,k}{\tilde{\Lambda }}_{M,k}{\tilde{U}}_{M,k}^{T}\right)}^{-1}, \end{array}$$

where *T*_*m*_^−1^ is calculated using the SMW formula according to the last result *T*_*m*−1_^−1^. After performing the series of *M*+1 operations, we can obtain ${\tilde{K}}^{-1}=T_{M}^{-1}$.

**Lemma 1 (see [33]).**

Let *A* and *C* both be invertible; then, Sherman–Morrison–Woodbury (SMW) formula gives an explicit formula for the inverse of matrices *A*+*UCV* if *C*^−1^+*VA*^−1^*U* is invertible.

(17) $$\begin{array}{c} {\left(A+UCV\right)}^{-1}=A^{-1}-A^{-1}U{\left(C^{-1}+VA^{-1}U\right)}^{-1}VA^{-1}. \end{array}$$

We can find that when the mixture weight is known, formula (15) is same as the dual problem of SVM. Hence, we have

(18) $$\begin{array}{c} J=\frac{1}{2}\alpha^{*T}Y\tilde{K}Y\alpha^{*}-e^{T}\alpha^{*}, \end{array}$$

where *α*^*∗*^ is the optimal solution minimizing (15). With *α*^*∗*^ considered a constant in *J*, *J* can be regarded as a function of **d**, and we calculate the gradient of the objective *J* with respect to *d*_*m*_.

(19) $$\begin{array}{c} \frac{\partial J}{\partial d_{m}}=\frac{1}{2}\alpha^{*T}Y\left({\tilde{U}}_{m,k}{\tilde{\Lambda }}_{m,k}{\tilde{U}}_{m,k}^{T}+\sigma I\right)Y\alpha^{*}\\ =\frac{1}{2}\alpha^{*T}Y\left({\tilde{U}}_{m,k}{\tilde{\Lambda }}_{m,k}{\tilde{U}}_{m,k}^{T}\right)Y\alpha^{*}+\frac{\sigma }{2}\alpha^{*T}\alpha^{*}. \end{array}$$

We use the reduce gradient method in [27] to deal with problem (14). First, for satisfying the *L*1-norm constraint on the weight vector **d** in (14), we calculate the reduced gradient of **d**:

(20) $$\begin{array}{c} {\left[\nabla_{\text{red}}J\right]}_{m}=\frac{\partial J}{\partial d_{m}}-\frac{\partial J}{\partial d_{\mu }},\forall m\neq \mu ,\mu =\underset{m}{\arg\max}d_{m},\\ {\left[\nabla_{\text{red}}J\right]}_{\mu }=\sum_{m\neq \mu }\left(\frac{\partial J}{\partial d_{\mu }}-\frac{\partial J}{\partial d_{m}}\right),\;\mu =\underset{m}{\arg\max}d_{m}, \end{array}$$

where ∇_red_*J* denotes the reduced gradient of *J*(**d**). Let *d*_*μ*_ be the largest element of the vector **d**, and *μ* be the corresponding index. Obviously, −∇_red_*J* would be a descent direction. However, if ∃*m* that makes *d*_*m*_=0 with −[∇_red_*J*]_*m*_ < 0, then *d*_*m*_⟶0^−^, which does not meet the nonnegative restriction. Therefore, −[∇_red_*J*]_*m*_ needs to be set to 0. Update descent direction is as follows:

(21) $$\begin{array}{c} D_{m}=\left\{\begin{array}{cc} 0, & d_{m}=0,{\left[\nabla_{\text{red}}J\right]}_{m}>0,\\ -{\left[\nabla_{\text{red}}J\right]}_{m}, & d_{m}>0,m\neq \mu ,\\ -{\left[\nabla_{\text{red}}J\right]}_{\mu }, & m=\mu . \end{array}\right) \end{array}$$

In general, MKL uses a two-step training method. It requires frequent calls to support vector machine solvers, which is prohibitive for large scale problems. Therefore, after each update on **d**, we are not eager to substitute it into support vector machine solvers to update *α*^*∗*^, but continue to look for the maximum allowable step length in this descent direction until the objective function value stops declining. Finally, we get the optimal step length by the line search method. The complete algorithm of the multi-Nyström method with MKL is summarized in Algorithm 1.

### 3.3. Kernel Stability Analysis

In some previous related works, Nyström is usually considered as a preprocessing method and mostly only study the approximate error bounds without considering the impact of the approximate on the performance of the kernel machine. In the following, we analyze the kernel stability of our method, bounding the relative performance based on the weighted kernel approximation error. It provides performance guarantees for our multi-Nyström approximate method in the context of large scale imbalanced classification.

**Proposition 1 .**

Let *α*^*∗*^ be the optimal solution for kernel SVM with kernel *K* and $\tilde{\alpha }$ be the solution of kernel SVM with kernel $\tilde{K}$ obtained by Nyström approximation. Then,

(22) $$\begin{array}{c} {\left\|\tilde{\alpha }-\alpha^{*}\right\|}_{2}\leq \theta^{2}\frac{1+{\left\|\tilde{K}\right\|}_{2}}{\lambda_{\min}}\Delta \;\text{with }\Delta =\sum_{m=1}^{M}d_{m}{\left\|\left({\tilde{K}}_{m}-K_{m}\right)\right\|}_{2}{\left\|\alpha^{*}\right\|}_{2}, \end{array}$$

where *λ*_min_ is the smallest eigenvalue of $\tilde{K}$, and *θ* is the constant from Hoffman's bound independent on *α*^*∗*^ and $\tilde{\alpha }$.

**Proof**

Define ∇^+^*f*(**x**) ≡ **x** − [**x** − ∇*f*(**x**)]_*𝒳*_^+^ be the projected gradient, where *𝒳* is the bounded constraint and [**x**]_*𝒳*_^+^ ≡ argmin_**y**∈*𝒳*_‖**x** − **y**‖ is the convex projection operator. It can be used to define an error bound according to the following theorem:

**Theorem 1 (see [34]).**

Let $\tilde{x}$ be the nearest optimal solution of the convex optimization problem:

(23) $$\begin{array}{c} \underset{x\in X}{\min}f\left(x\right)=g\left(Ex\right)+b^{T}x, \end{array}$$

with *g*(**t**) being *σ*_*g*_ strongly convex, ∇*f*(**x**) being *ρ* Lipschitz continuous, and *𝒳*={**x***|A ***x** ≤ *d*} is a polyhedral set. The optimization problem admits a global error bound:

(24) $$\begin{array}{c} \left\|x-\tilde{x}\right\|\leq \theta^{2}\frac{1+\rho }{\sigma_{g}}\left\|\nabla^{+}f\left(x\right)\right\|,\;\forall x\in X, \end{array}$$

where *θ* is the constant from Hoffman's bound.

Considering now the problem $\min_{\alpha \in \Omega }\tilde{f}\left(\alpha \right)=\tilde{g}\left(C^{T}Y\alpha \right)\left(\right)-e^{T}\alpha$ with $\tilde{g}\left(x\right)=\left(1/2\right)x^{T}W_{k}^{+}x$ and bounded constraint Ω={*α| ***y**^*T*^*α*=0,  0 ≤ *α* ≤ *C*}, then

(25) $$\begin{array}{c} \underset{\alpha \in \Omega }{\min}\tilde{f}\left(\alpha \right)=\frac{1}{2}\alpha^{T}YCW_{k}^{+}C^{T}Y\alpha -e^{T}\alpha . \end{array}$$

Note that the above problem is equivalent to problem (15) with the equality $\tilde{K}=CW_{k}^{+}C^{T}$ (*W*_*k*_^+^ is SPSD), and we have

(26) $$\begin{array}{c} \lambda_{\min}\left(W_{k}^{+}\right){\left\|x-y\right\|}^{2}\leq {\left(\nabla \tilde{g}\left(x\right)-\nabla \tilde{g}\left(y\right)\right)}^{T}\left(x-y\right)\\ ={\left(x-y\right)}^{T}W_{k}^{+}\left(x-y\right)\\ ⟹\sigma_{\bar{g}}=\lambda_{\min}\left(W_{k}^{+}\right),\\ \left\|\nabla \tilde{f}\left(x\right)-\nabla \tilde{f}\left(y\right)\right\|=\left\|YCW_{k}^{+}C^{T}Y\left(x-y\right)\right\|\\ \leq \left\|CW_{k}^{+}C^{T}\right\|\left\|x-y\right\|\\ =\left\|\tilde{K}\right\|\left\|x-y\right\|\\ ⟹\rho ={\left\|\tilde{K}\right\|}_{2}. \end{array}$$

Let *f* be the dual objective function of multiple kernel learning problem (5) with the original kernel *K*=∑_*m*_*d*_*m*_*K*_*m*_, and $\tilde{f}$ be the objective function of approximate multiple kernel learning problem (9) with kernel $\tilde{K}=\sum_{m}d_{m}{\tilde{K}}_{m}$ obtained by our multi-Nyström method (13). Consider now *α*^*∗*^ and $\tilde{\alpha }$ as the optimal solutions of *f*(*α*) and $\tilde{f}\left(\alpha \right)$, respectively. We have

(27) $$\begin{array}{c} \nabla \tilde{f}\left(\alpha^{*}\right)=Y\tilde{K}Y\alpha^{*}-YKY\alpha^{*}+YKY\alpha^{*}-e\\ =Y\left(\tilde{K}-K\right)Y\alpha^{*}+\nabla f\left(\alpha^{*}\right), \end{array}$$

where we use the fact that $\nabla f\left(\alpha^{*}\right)=0\text{ and }\nabla \tilde{f}\left(\tilde{\alpha }\right)=0$; therefore,

(28) $$\begin{array}{c} {\left\|\nabla \tilde{f}\left(\alpha^{*}\right)\right\|}_{2}={\left\|\sum_{m=1}^{M}d_{m}Y\left({\tilde{K}}_{m}-K_{m}\right)Y\alpha^{*}\right\|}_{2}\\ \leq \sum_{m=1}^{M}d_{m}{\left\|\left({\tilde{K}}_{m}-K_{m}\right)\alpha^{*}\right\|}_{2}\\ \leq \sum_{m=1}^{M}d_{m}{\left\|\left({\tilde{K}}_{m}-K_{m}\right)\right\|}_{2}{\left\|\alpha^{*}\right\|}_{2}, \end{array}$$

where ${\left\|\left({\tilde{K}}_{m}-K_{m}\right)\right\|}_{2}$ is the spectral norm error of the *m*^th^ Nyström approximate based on the *m*^th^ subset of landmark points.

Furthermore, we use the inequality ${\left\|\nabla^{+}\tilde{f}\left(\alpha^{*}\right)\right\|}_{2}\leq {\left\|\nabla \tilde{f}\left(\alpha^{*}\right)\right\|}_{2}$ of the kernel SVM given by [35] (proof of Theorem 2) along with Theorem 1 to upper bound the norm difference between the optimal solutions of *f*(*α*) and $\tilde{f}\left(\alpha \right)$:

(29) $$\begin{array}{c} {\left\|\alpha^{*}-\tilde{\alpha }\right\|}_{2}\leq \theta^{2}\frac{1+\rho }{\sigma_{\tilde{g}}}{\left\|\nabla^{+}\tilde{f}\left(\alpha^{*}\right)\right\|}_{2}\\ \leq \theta^{2}\frac{1+\rho }{\sigma_{\tilde{g}}}{\left\|\nabla \tilde{f}\left(\alpha^{*}\right)\right\|}_{2}\\ \leq \theta^{2}\frac{1+\rho }{\sigma_{\tilde{g}}}\sum_{m=1}^{M}d_{m}{\left\|\left({\tilde{K}}_{m}-K_{m}\right)\right\|}_{2}{\left\|\alpha^{*}\right\|}_{2}. \end{array}$$

The proposition shows us the norm difference ${\left\|\alpha^{*}-\tilde{\alpha }\right\|}_{2}$ is controlled by a weighted Nyström approximate error. And it guides us to focus on approximating the kernel matrices with greater weights for getting a better learning performance.

## 4. Experiments

In this section, in order to validate the efficiency of the proposed method in solving large scale imbalanced problems, we compare our method against kernel methods including SVM and MKSVM (multiple kernel SVM), as well as the Nyström approximation method. All experiments are implemented on a PC with Intel quad-core i7-8565U CPU@1.80 GHz and 8 GB memory.

### 4.1. Implementation

We implement our experiments on five real-world imbalanced datasets from the KEEL data repository (https://keel.es/) and the LIBSVM archive (https://www.csie.ntu.edu.tw/cjlin/libsvmtools/datasets/) (Table 1). For a fair comparison, we perform 10 times stratified 5-fold cross-validation and report the average result. We use LIBSVM (https://www.csie.ntu.edu.tw/cjlin/libsvm/index.html) and SimpleMKL (https://asi.insa-rouen.fr/enseignants/arakoto/code/mklindex.html) to run kernel SVM and MKSVM, respectively. As the kernel type, all experiments use the Gaussian kernel with bandwidth *σ* in the range of log_10_  *σ*={−1,0,1,2}. Because we are interested in relative performance, we empirically set the trade-off parameter *C* = 100. In this study, we adopt the following three evaluation measures of the classification performance on imbalanced datasets: *F*1 score, *G*-mean, and area under ROC curve (AUC).

(30) $$\begin{array}{c} \text{PRE}=\frac{\text{TP}}{\text{TP}+\text{FP}},\\ \text{REC}=\frac{\text{TP}}{\text{TP}+\text{FN}},\\ \text{SPE}=\frac{\text{TN}}{\text{TN}+\text{FP}},\\ F1\text{ score}=\frac{2\times \text{PRE}\times \text{REC}}{\text{PRE}+\text{REC}},\\ G-\text{mean}=\sqrt{\text{REC}\times \text{SPE}}, \end{array}$$

where TP, TN, FP, and FN represent the number of true-positive, true-negative, false-positive, and false-negative instances, respectively. *F*1 score measures the classification performance on the minority class. *G*-mean reflects the overall classification performance. AUC works well for comparing performance between algorithms [36].

### 4.2. Experimental Results

Table 2 provides the average experimental results of the proposed method and the other three algorithms on the four imbalanced datasets using the above three measures. We first compare SVM and the standard Nyström method. The Nyström method uses uniform sampling without replacement to approximate the kernel matrix, which relieves the model's sensitivity to class imbalance to a certain extent. For example, on the Poker-8-9_vs_5 dataset, in terms of G-mean, the Nyström method improves nearly 7 times more than SVM. However, we can also see that in terms of AUC and *F*1 score, there still exits a large gap in model accuracy as compared with SVM.

Next, we compare our multi-Nyström method with the standard Nyström method. The experimental results clearly demonstrate that our method outperforms the Nyström method, especially in the context of extreme imbalance. This mainly benefits from the use of undersampling of the majority class, which can effectively balance the class distribution. Moreover, it can be seen that multi-Nyström can improve the accuracy of the model. For example, with the same number of landmark points, the *F*1 score and AUC value of multi-Nyström on the USPS dataset are closer to that of SVM or even higher on Poker-8-9_vs_5 and Page-blocks0 datasets.

Note that our method is also a type of approximation of MKL, and finally, we also examine the performance of MKL-based MKSVM. From the results, we can see the effect of using MKL to represent input data, which also implicitly explains how our method achieves better accuracy at the expense of more computations.

### 4.3. Discussion

**Figure 2:**
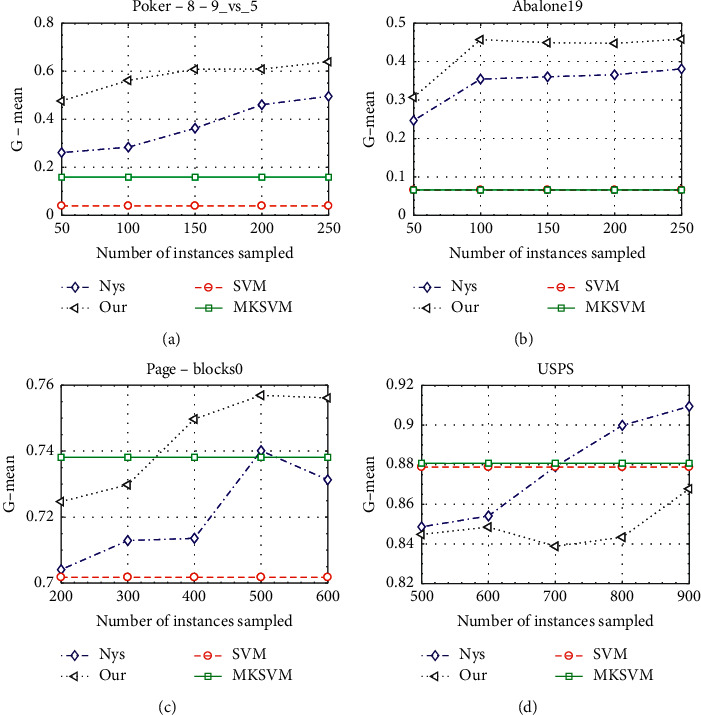
Classification performance with different numbers of instances sampled on four datasets.

In this part, we further discuss the impact of different parameters on performance. In the first experiment, in order to study the impact of the number of sampling landmark points on the classification performance, we fix the approximate rank parameter and successively increase the number of sampling landmark points, and then train and test the SVM model on four datasets, with results as shown in Figure 2. We can see that as the number of sampling landmark points increases, although there are some fluctuations, the performance of our method and Nyström still presents a rising trend. Moreover, except for few cases, our method uses fewer landmark points and can still yield higher G-mean.

**Figure 3:**
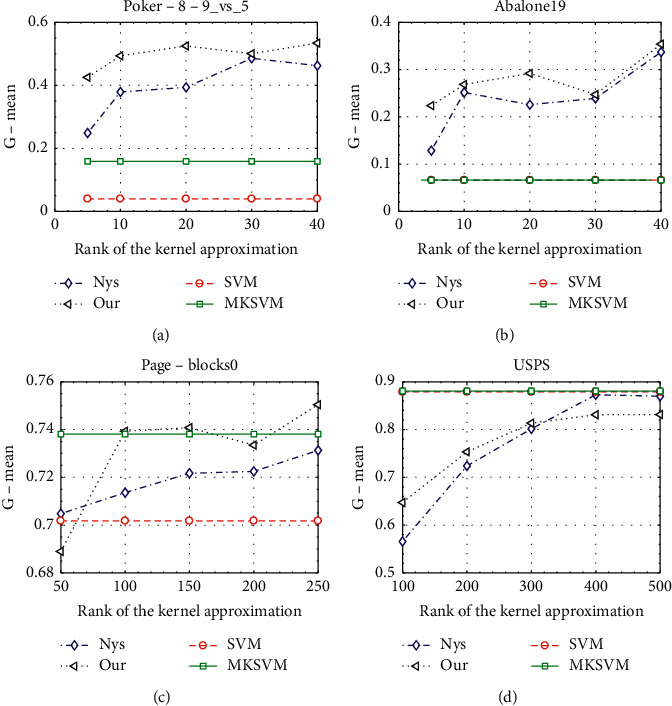
Classification performance with different ranks of the kernel approximation on four datasets.

In the second experiment, we study the performance with the variance of the rank parameter.  Figure 3 shows the *G*-mean on four datasets by varying the approximate rank. They show us that with the same approximate kernel rank, our method can achieve better classification performance than others.

**Figure 4:**
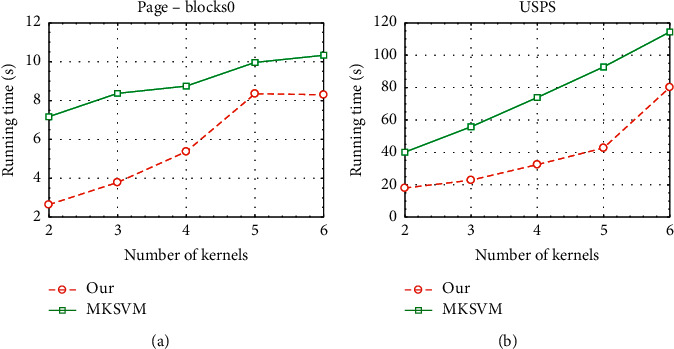
Timing performance with different numbers of kernels on two datasets.

Finally, we further compare the running time of our method and MKSVM. We report the results on two datasets USPS and Page-blocks in Figure 4. The results show that our method can significantly speedup the MKL process under guaranteed performance. For example, on the USPS dataset, our method can reduce the running time by more than one order of magnitude. The main reason is due to the low-rank attribute of the approximate kernel matrix that speeds up the MKL algorithm process.

**Figure 5:**
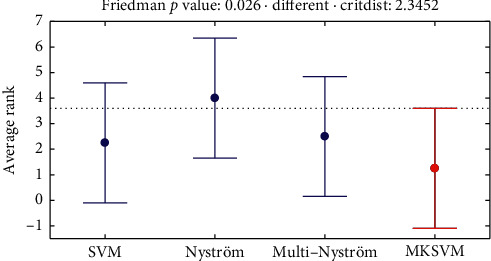
Average rank of the four algorithms for four datasets.

For further analysis of the experimental results, we perform the Friedman test with respect to the *F*1 score. First, we calculate the average ranks of SVM, Nyström, multi-Nyström, and MKSVM as shown in Figure 5. It can be noticed that MKSVM gives the best performance. Meanwhile, the SVM and the proposed multi-Nyström rank similarly. In a comparison of *k* algorithms on *N* datasets, considering *r*_*i*_ as the average ranking of the *i* ^th^ algorithm, the Friedman variable *F*_*F*_ can be calculated as follows:

(31) $$\begin{array}{c} F_{F}=\frac{\left(N-1\right)\chi_{F}^{2}}{N\left(k-1\right)-\chi_{F}^{2}}, \end{array}$$

with

(32) $$\begin{array}{c} \chi_{F}^{2}=\frac{12N}{k\left(k+1\right)}\left(\sum_{i=1}^{k}r_{i}^{2}-\frac{k{\left(k+1\right)}^{2}}{4}\right), \end{array}$$

where *F*_*F*_ is distributed to (4 − 1) and (4 − 1)(4 − 1) degrees of freedom. For our experiments, *F*_*F*_=10.3333. The critical value of *F*(3,9) is 3.8625 for *α*=0.05. Since *F*_*F*_ > *F*(3,9), we can reject the null hypothesis that all the algorithms have the same performance. Then, we perform the Nemenyi test to compare algorithms pairwise. The critical difference is calculated as follows:

(33) $$\begin{array}{c} \text{CD}=q_{a}\sqrt{\frac{k\left(k+1\right)}{6N}}, \end{array}$$

considering *α*=0.05 and CD=2.3452. The difference between the average ranking of the SVM, Nyström, and multi-Nyström with MKSVM is 1.0, 2.75, and 1.25, respectively. Hence, we can state that the best MKSVM is significantly better than Nyström at *α*=0.05. However, the difference between the best MKSVM and the proposed multi-Nyström is not significant, which indicates the proposed method achieves better performance than the standard Nyström kernel classifier and more efficiency than the best MKSVM.

## 5. Conclusions

In this study, we propose a novel method to overcome the time and memory limitations of the standard Nyström method and extend it to the case of large scale imbalanced classification. In general, kernel approximation and model training are carried out separately. To obtain more accurate results, our method mixes multiple Nyström approximations and embeds them in the model training process to learn the model parameters and mixture weights simultaneously. In particular, the approximate kernel matrix yielded by our method is low rank and balanced. We also provide an error bound of the model solution based on our approximate method to guide us in improving the learning process. Experimental results show that our method can achieve a higher classification accuracy. On the other hand, it can dramatically improve the efficiency of exiting MKL algorithms.

Potential improvements: there are still some caveats in our current solution. For example, due to the curse of kernelization, the number of support vectors grows in an unbounded manner when suffered the nonzero loss. This significantly increases the computational cost and can be infeasible for large scale problems. Future work will chiefly focus on more efficient variants of multi-Nyström involving budget kernel learning to address the issue.

## Figures and Tables

**Algorithm 1:**
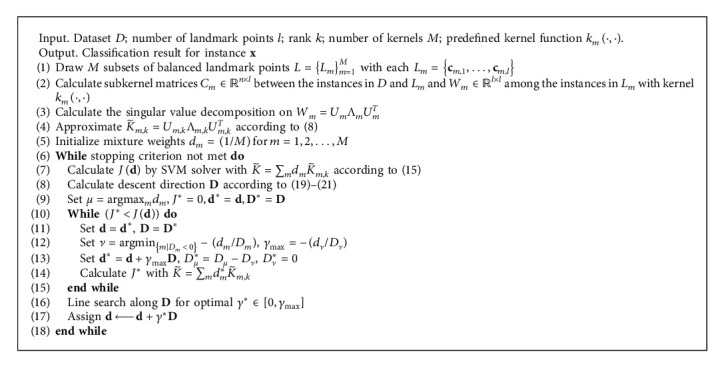
The proposed MKLMO algorithm.

**Table 1:** Datasets used in experiments

Dataset	# feature	# instance	IR
Poker-8-9_vs_5	10	2075	82
Abalone19	8	4174	129.44
Page-blocks0	10	5472	8.79
USPS (class 9 against all)	256	9298	12.13

**Table 2:** *F*1 score, *G*-mean, and AUC results of different algorithms on four datasets

Datasets	Measures	SVM	Nyström	Multi-Nyström	MKSVM
Poker-8-9_vs_5	*F*1	0.0571	0.0327	0.0585	0.1906
	*G*-mean	0.0399	0.3357	0.5140	0.1589
	AUC	0.8107	0.6106	0.7953	0.7942

Abalone19	*F*1	0.0611	0.0200	0.0334	0.0569
	*G*-mean	0.0661	0.2914	0.3500	0.0661
	AUC	0.7487	0.5203	0.6094	0.7263

Page-blocks0	*F*1	0.8061	0.7954	0.8171	0.8342
	*G*-mean	0.7018	0.7292	0.7115	0.7381
	AUC	0.9857	0.9753	0.9585	0.9904

USPS	*F*1	0.8991	0.6688	0.8853	0.9102
	*G*-mean	0.8788	0.8593	0.8408	0.8807
	AUC	0.9939	0.9608	0.9874	0.9963

## Data Availability

The data used to support the findings of this study have been deposited in the KEEL repository (http://keel.es/) and the LIBSVM archive (https://www.csie.ntu.edu.tw/cjlin/libsvmtools/datasets/).

## References

[1] Huang C., Li Y., Change Loy C., Tang X. Learning deep representation for imbalanced classification.

[2] Zhu J., Hovy E. Active learning for word sense disambiguation with methods for addressing the class imbalance problem.

[3] Sun W., Sun J., Zhu Y., Zhang Y. (2020). Video super-resolution via dense non-local spatial-temporal convolutional network. *Neurocomputing*.

[4] Chawla N. V. (2009). Data mining for imbalanced datasets: an overview. *Data Mining and Knowledge Discovery Handbook*.

[5] Luo X., Sun J., Wang L. (2018). Short-term wind speed forecasting via stacked extreme learning machine with generalized correntropy. *IEEE Transactions on Industrial Informatics*.

[6] Sun Y., Wong A. K. C., Kamel M. S. (2009). Classification of imbalanced data: a review. *International Journal of Pattern Recognition and Artificial Intelligence*.

[7] Galar M., Fernandez A., Barrenechea E., Bustince H., Herrera F. (2011). A review on ensembles for the class imbalance problem: bagging-, boosting-, and hybrid-based approaches. *IEEE Transactions on Systems, Man, and Cybernetics, Part C (Applications and Reviews)*.

[8] Haixiang G., Yijing L., Shang J., Mingyun G., Yuanyue H., Bing G. (2017). Learning from class-imbalanced data: review of methods and applications. *Expert Systems with Applications*.

[9] Chawla N. V., Bowyer K. W., Hall L. O., Kegelmeyer W. P. (2002). Smote: synthetic minority over-sampling technique. *Journal of Artificial Intelligence Research*.

[10] He H., Bai Y., Edwardo Garcia A., Li S. Adasyn: adaptive synthetic sampling approach for imbalanced learning.

[11] Batuwita R., Palade V. (2010). FSVM-CIL: fuzzy support vector machines for class imbalance learning. *IEEE Transactions on Fuzzy Systems*.

[12] Yu H., Sun C., Yang X., Zheng S., Zou H. (2019). Fuzzy support vector machine with relative density information for classifying imbalanced data. *IEEE Transactions on Fuzzy Systems*.

[13] Hong X., Chen S., Harris C. J. (2007). A kernel-based two-class classifier for imbalanced data sets. *IEEE Transactions on Neural Networks*.

[14] Tang Y., Zhang Y.-Q., Chawla N. V., Krasser S. (2008). “SVMS modeling for highly imbalanced classification. *IEEE Transactions on Systems, Man, and Cybernetics, Part B (Cybernetics)*.

[15] Maratea A., Petrosino A., Manzo M. (2014). Adjusted F-measure and kernel scaling for imbalanced data learning. *Information Sciences*.

[16] Mathew J., Khiang Pang C., Luo M., Leong W. H. (2017). Classification of imbalanced data by oversampling in kernel space of support vector machines. *IEEE Transactions on Neural Networks and Learning Systems*.

[17] Wu G., Chang E. Y. (2005). KBA: kernel boundary alignment considering imbalanced data distribution. *IEEE Transactions on Knowledge and Data Engineering*.

[18] Tang Bo, He H. Kerneladasyn: kernel based adaptive synthetic data generation for imbalanced learning.

[19] Williams C., Seeger M. (2000). Using the Nyström method to speed up kernel machines. *Advances in Neural Information Processing Systems*.

[20] Drineas P., Mahoney M. W. (2005). On the Nyström method for approximating a gram matrix for improved kernel-based learning. *Journal of Machine Learning Research*.

[21] Musco C., Musco C. Recursive sampling for the Nyström method.

[22] Kumar S., Mohri M., Talwalkar A. (2009). Ensemble Nyström method. *Advances in Neural Information Processing Systems*.

[23] Li Z., Yang T., Zhang L., Jin R. Fast and accurate refined Nyström-based kernel SVM.

[24] Hofmann T., Schölkopf B., Smola A. J. (2008). Kernel methods in machine learning. *The Annals of Statistics*.

[25] Vapnik V. (2013). *The Nature of Statistical Learning Theory*.

[26] Lanckriet G. R. G., Cristianini N., Bartlett P., El Ghaoui L., Jordan M. I. (2004). Learning the kernel matrix with semidefinite programming. *Journal of Machine Learning Research*.

[27] Rakotomamonjy A., Bach F. R., Canu S., Grandvalet Y. (2008). Simplemkl. *Journal of Machine Learning Research*.

[28] Alioscha-Perez M., Cédric Oveneke M., Sahli H. (2019). SVRG-MKL: a fast and scalable multiple kernel learning solution for features combination in multi-class classification problems. *IEEE Transactions on Neural Networks and Learning Systems*.

[29] Kumar S., Mohri M., Talwalkar A. Sampling techniques for the Nyström method.

[30] Wang L., Wang H., Fu G. (2021). Multiple kernel learning with minority oversampling for classifying imbalanced data. *IEEE Access*.

[31] Gönen M., Alpaydın E. (2011). Multiple kernel learning algorithms. *The Journal of Machine Learning Research*.

[32] Kumar S., Mohri M., Talwalkar A. (2012). Sampling methods for the Nyström method. *The Journal of Machine Learning Research*.

[33] Deng C. Y. (2011). A generalization of the Sherman-Morrison-Woodbury formula. *Applied Mathematics Letters*.

[34] Wang P.-W., Lin C.-J. (2014). Iteration complexity of feasible descent methods for convex optimization. *The Journal of Machine Learning Research*.

[35] Hsieh C.-J., Si Si, Dhillon I. S. Fast prediction for large-scale kernel machines.

[36] Barua S., Monirul Islam Md, Yao X., Murase K. (2012). Mwmote–majority weighted minority oversampling technique for imbalanced data set learning. *IEEE Transactions on Knowledge and Data Engineering*.

